# Clinical inertia in type 2 diabetes management in a middle-income country: A retrospective cohort study

**DOI:** 10.1371/journal.pone.0240531

**Published:** 2020-10-09

**Authors:** Kim Sui Wan, Foong Ming Moy, Khalijah Mohd Yusof, Feisul Idzwan Mustapha, Zainudin Mohd Ali, Noran Naqiah Hairi

**Affiliations:** 1 Centre for Epidemiology and Evidence-Based Practice, Department of Social and Preventive Medicine, Faculty of Medicine, University of Malaya, Kuala Lumpur, Malaysia; 2 State Health Department of Negeri Sembilan, Seremban, Malaysia; 3 Disease Control Division, Ministry of Health, Putrajaya, Malaysia; Shanghai Diabetes Institute, CHINA

## Abstract

**Background:**

Clinical inertia can lead to poor glycemic control among type 2 diabetes patients. However, there is paucity of information on clinical inertia in low- and middle-income countries including Malaysia. This study aimed to determine the time to treatment intensification among T2D patients with HbA1c of ≥7% (≥53 mmol/mol) in Malaysian public health clinics. The proportion of patients with treatment intensification and its associated factors were also determined.

**Material and methods:**

This was a five-year retrospective open cohort study using secondary data from the National Diabetes Registry. The study setting was all public health clinics (n = 47) in the state of Negeri Sembilan, Malaysia. Time to treatment intensification was defined as the number of years from the index year until the addition of another oral antidiabetic drug or initiation of insulin. Life table survival analysis based on best-worst case scenarios was used to determine the time to treatment intensification. Discrete-time proportional hazards model was fitted for the factors associated with treatment intensification.

**Results:**

The mean follow-up duration was 2.6 (SD 1.1) years. Of 7,646 patients, the median time to treatment intensification was 1.29 years (15.5 months), 1.58 years (19.0 months) and 2.32 years (27.8 months) under the best-, average- and worst-case scenarios respectively. The proportion of patients with treatment intensification was 45.4% (95% CI: 44.2–46.5), of which 34.6% occurred only after one year. Younger adults, overweight, obesity, use of antiplatelet medications and poorer HbA1c were positively associated with treatment intensification. Patients treated with more oral antidiabetics were less likely to have treatment intensification.

**Conclusion:**

Clinical inertia is present in the management of T2D patients in Malaysian public health clinics. We recommend further studies in lower- and middle-income countries to explore its causes so that targeted strategies can be developed to address this issue.

## Introduction

The importance of glycemic control in patients with type 2 diabetes (T2D) is well established [1]. Clinical practice guidelines recommend frequent monitoring of HbA1c (e.g. every three months) with stepwise treatment intensification (TI) until glycemic target is achieved [2, 3]. However, glycemic control was often found to be inadequate. In some European countries and the United States of America (USA), 53.6% and 41.9% of T2D patients respectively achieved HbA1c goal of <7% (<53 mmol/mol) [4, 5]. In Malaysia, an upper-middle-income country, only 37.9% achieved the same glycemic target [6].

Patients’ non-adherence to treatment and clinical inertia are two main reasons for suboptimal glycemic control [7, 8]. Clinical inertia is defined as the ‘failure of healthcare providers to initiate or intensify therapy when indicated’ [9]. Most studies quantify clinical inertia by measuring the proportion of patients with suboptimal HbA1c who received TI within a specific time frame [10]. Some researchers measure clinical inertia as the median time to TI, after at least one HbA1c reading was above a certain threshold [10]. The reasons for clinical inertia are multifactorial and often arises from a complex interplay between patient-, clinician-, and health system-related factors [8].

The median time to TI was more than one year in most of the studies reviewed in a systematic review [10]. A study from the USA reported that almost two-thirds of T2D patients on two oral antidiabetic drugs with HbA1c of ≥7% (≥53 mmol/mol) had no evidence of TI. Moreover, the proportions of patients with no TI was 53.3% and 44.4% for HbA1c between 8% and 8.9% (64–74 mmol/mol) and HbA1c of ≥9.0% (≥75 mmol/mol) respectively [11].

The evidence on clinical inertia in T2D management is scarce in low- and middle-income countries (LMIC) including Malaysia [10]. Hence, this study aimed to determine the time to TI among T2D patients with HbA1c of ≥7% (≥53 mmol/mol). In addition, the proportion and factors associated with TI were also determined.

## Materials and methods

### Study design

This was a five-year retrospective open cohort study conducted from the year 2013 to 2017. The study setting was all public health clinics (n = 47) in the state of Negeri Sembilan, Malaysia. Malaysia, with an estimated population of 32.7 million, is a multi-ethnic country with Bumiputra (including Malays), Chinese, Indians and other ethnicities [12]. Negeri Sembilan is located to the south of capital city Kuala Lumpur. Almost 60% of diabetic patients in Malaysia received treatment from public health clinics [13]. Study participants were T2D patients, aged 18 years and above, had HbA1c of ≥7% (≥53 mmol/mol) and not treated with insulin in the index year. In addition, only patients who had at least two clinical audits between the year 2013 and 2017 were selected. Patients with other types of diabetes were excluded.

### Data source

Secondary data from the National Diabetes Registry of Malaysia, a web-based diabetes surveillance platform was used in this study [6]. Clinical audits were conducted in August annually, whereby T2D patients from all public health clinics were randomly sampled. All active patients had an equal probability to be sampled regardless of whether they had been audited before. Within each year, patients would have several visits to the clinic. Patients’ last observed clinical and laboratory results for the year were selected and entered in the web-based registry to represent the whole year performance [6]. The one-year audit timeframe occurred from 1^st^ August to 31^st^ July in the subsequent year. Hence, the audited data were discrete-time (interval-censored) data i.e. the information was known to occur within a one-year interval but without knowing the exact date. This five-year study period occurred between 1 August 2012 and 31 July 2017.

The diagnosis of T2D was based on venous plasma glucose (≥7.0 mmol/L for fasting or ≥11.1 mmol/L for random) or HbA1c ≥6.3% (45 mmol/mol). In symptomatic individual, one abnormal value was diagnostic whereas in asymptomatic individual, two abnormal values were required [3]. Baseline information on demographic characteristics, smoking status, comorbidities, diabetes complications and treatment profiles were collected. The demographic factors were age, sex and ethnicity. Age was categorized into younger adults aged below 60 years and older adults aged at least 60 years based on the United Nations’ definition [14]. Smoking status was yes/no to current smoking. The comorbidities were overweight/obesity based on the World Health Organization classification [15]; dyslipidemia as diagnosed clinically or with the use of lipid-lowering medication; and hypertension as diagnosed clinically or with the use of antihypertensive medication [16]. Diabetes-related complications such as ischemic heart disease, stroke, retinopathy, nephropathy and foot complication were based on clinical diagnoses [16]. Treatment profiles included information on number of oral antidiabetic drugs (OADs), use of antihypertensive, lipid-lowering and antiplatelet medications. Other variables included were duration of diabetes and polypharmacy which was the use of five or more types of medications [17].

The outcome of interest was time to TI, calculated as the number of years from the index year until TI occurred by an increase in the number of OAD or the initiation of insulin. Timely TI was defined as TI that occurred within one year. The one-year cutoff was to account for the time needed to titrate a pre-existing OAD regimen to the maximum dosage; after which, TI should occur as clinically indicated.

**Fig 1:**
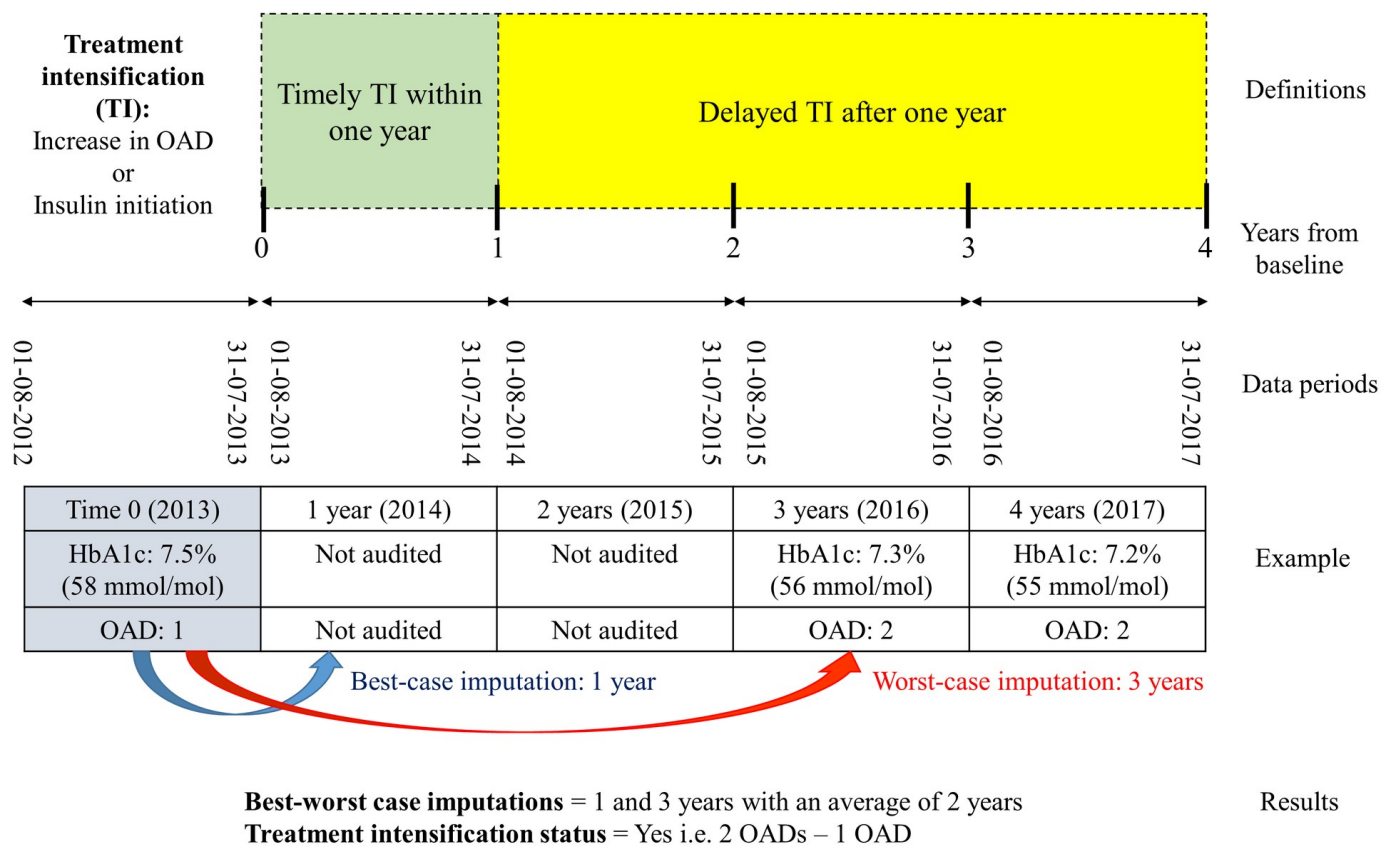
Definitions and an example using best- and worst-case scenario analysis.

As individual patients were randomly and independently sampled for the clinical audit each year, there were missing data in between these two audited years. The nature of these missing data was missing completely at random (MCAR) and therefore do not lend bias to the observed data [18]. We handled this missing data by using best-, average- and worst-case scenarios to calculate the time to TI. Similar approach of best- and worst-case scenario analysis had been used to impute outcomes and recreate the most extreme possible datasets in meta-analysis of clinical trials [19]. For example, if a patient was audited in the index year 2013 and data was missing in 2014 and 2015 before TI was observed in 2016, the TI could have happened either within a year in 2014, between one-to-two years in 2015 or between two-to-three years in 2016. Hence, one and three years were taken as the best- and worst-case scenarios respectively with the average time of two years (Fig 1).

There were no missing data in determining the TI status, as all patients in this study had at least two observed values. We assumed there was no change in the treatment regimen within these intervals. De-intensification of treatment was unlikely because this study population had uncontrolled HbA1c. In addition, treatment de-intensification was uncommon even in older T2D patients who were at high-risk of hypoglycemia [20].

### Statistical analyses

Baseline characteristics were described for the total population and by TI status. These were presented as number (%) for the categorical variables and mean (standard deviation, SD) or median (interquartile range, IQR) for the continuous variables. Independent t-test was used to compare the means while the Mann-Whitney test was used to compare the medians between patients with and without TI.

The time to TI for discrete-time data was described using life table survival analysis for the best-, average- and worst-case scenarios [21]. Interpolation was used to compare the median time to TI which was most useful when both the values were found within the same time interval [21]. Hence, the plotted graph showed a series of joined line segments instead of step functions [21]. Censoring occurred when no TI achieved at the end of the study, when patients exited the cohort (due to loss to follow-up, death or were not subsequently sampled for clinical audit) and when the HbA1c value fell below 7% (53 mmol/mol).

The proportion of the study population with TI was presented as number (%) with a 95% confidence interval, CI. For univariate analysis, Pearson chi-square tests were used to compare the proportions of patients with and without TI. For multivariate analysis, we used complementary log-log transformation to yield a proportional hazards model [21]. This provided a discrete-time analog for the continuous-time Cox proportional hazards model [21]. Maximum likelihood estimation and Wald chi-square statistics were used to fit the multivariate proportional hazards model. Log likelihood, Akaike’s information criterion (AIC) and Bayesian information criterion (BIC) were used to assess the model fit. Statistical significance was preset at p <0.05. Hazards ratio with 95% confidence intervals were reported.

We performed sensitivity analysis by using HbA1c cutoffs of ≥8% (≥64 mmol/mol) and ≥9% (≥75 mmol/mol) to explore whether HbA1c levels would affect the time to TI and the proportion of patients with TI. In addition, we repeated the analyses for a subgroup of patients with HbA1c above individualized target of below 8% (64 mmol/mol) [22]. We defined individualized HbA1c treatment target of below 8% (64 mmol/mol) for patients with three or more comorbidities/complications, had long-standing diabetes for more than 20 years or aged 75 years and above [3, 23]. The average life expectancy in Malaysia was 74.5 years [24].

To understand how the number of OADs affected the median time to TI, we performed a post-hoc life table analysis under the best- and worst-case scenarios. We also compared the characteristics between several ethnicities to investigate factors leading to low TI among Chinese patients. We used parametric one-way ANOVA and non-parametric Kruskal-Wallis tests for continuous variables. For categorical variables, we used Pearson chi-square test; Fischer’s exact test was used when more than 20% of the cells had expected frequencies below five. All analyses were conducted using the IBM SPSS Statistical Software 23.

### Ethics approval

This study was approved by the Malaysia Medical Research Ethics Committee (MREC), reference number NMRR-18-2731-44032. Permission to collate the data was sought from the State Health Department of Negeri Sembilan. Written informed consent was not required in accordance with local legislation and national guidelines due to the retrospective nature of this study.

## Results

### Characteristics of all patients and by treatment intensification status

Table 1 shows the characteristics of all patients and by TI status. Among 7,646 eligible T2D patients, 55.6% were younger adults, 60.5% females, 65.5% Malays and 94.0% non-smoker. Their mean HbA1c was 8.1% (SD 1.6%). Comorbidities among patients were high as over 70% were overweight or obese, 80.4% had comorbid hypertension and 76.6% had dyslipidemia. About two-third of patients were treated with dual or triple OADs while 27.8% were on antiplatelet medications. Presence of polypharmacy was observed in 40.2% of patients. Diabetes complications occurred in 0.5% to 4.1% of patients.

**Table 1:** Characteristics of all patients and by treatment intensification status.

Characteristics		Total	Treatment intensification		*P value*
		n (column %)	Yes, n (row %)	No, n (row %)	
		7,646 (100)	3,469 (45.4)	4,177 (54.6)	
**Age,** mean (SD)		58.1 (10.3)	56.9 (10.1)	59.1 (10.4)	<0.001
	Younger adults	4,249 (55.6)	2,096 (49.3)	2,153 (50.7)	<0.001
	Older adults	3,397 (44.4)	1,373 (40.4)	2,024 (59.6)	
**Sex**					
	Male	3,021 (39.5)	1,370 (45.3)	1,651 (54.7)	0.976
	Female	4,625 (60.5)	2,099 (45.4)	2,526 (54.6)	
**Ethnicity**					
	Malay	5,010 (65.5)	2,371 (47.3)	2,639 (52.7)	<0.001
	Chinese	1,149 (15.0)	459 (39.9)	690 (60.1)	
	Indian	1,444 (18.9)	616 (42.7)	828 (57.3)	
	Others	43 (0.6)	23 (53.5)	20 (46.5)	
**Duration of diabetes**, median (IQR)		5.0 (6.0)	5.0 (6.0)	4.0 (6.0)	0.486
	<5 years	3,821 (50.0)	1,724 (45.1)	2,097 (54.9)	0.907
	5–10 years	2,746 (35.9)	1,253 (45.6)	1,493 (54.4)	
	>10 years	1,079 (14.1)	492 (45.6)	587 (54.4)	
**Smoker**					
	Yes	457 (6.0)	218 (47.7)	239 (52.3)	0.302
	No	7,189 (94.0)	3,251 (45.2)	3,938 (54.8)	
**Body mass index**, kg/m^2^, mean (SD) (n = 7,581 due to missing data)		28.1 (5.2)	28.5 (5.3)	27.8 (5.2)	<0.001
	Underweight, <18.5	74 (1.0)	26 (35.1)	48 (64.9)	<0.001
	Normal, 18.5 - <25.0	2,109 (27.6)	864 (41.0)	1,245 (59.0)	
	Overweight, 25 - <30.0	3,027 (39.6)	1,390 (45.9)	1,637 (54.1)	
	Obese, ≥30.0	2,371 (31.0)	1,170 (49.3)	1,201 (50.7)	
**Hypertension**					
	Yes	6,148 (80.4)	2,764 (45.0)	3,384 (55.0)	0.142
	No	1,498 (19.6)	705 (47.1)	793 (52.9)	
**Dyslipidemia**					
	Yes	5,858 (76.6)	2,652 (45.3)	3,206 (54.7)	0.754
	No	1,788 (23.4)	817 (45.7)	971 (54.3)	
**Ischemic heart disease**					
	Yes	201 (2.6)	99 (49.3)	102 (50.7)	0.262
	No	7,445 (97.4)	3,370 (45.3)	4,075 (54.7)	
**Stroke**					
	Yes	55 (0.7)	28 (50.9)	27 (49.1)	0.408
	No	7,591 (99.3)	3,441 (45.3)	4,150 (54.7)	
**Nephropathy**					
	Yes	316 (4.1)	135 (42.7)	181 (57.3)	0.334
	No	7,330 (95.9)	3,334 (45.5)	3,996 (54.5)	
**Retinopathy**					
	Yes	174 (2.3)	78 (44.8)	96 (55.2)	0.884
	No	7,472 (97.7)	3,391 (45.4)	4,081 (54.6)	
**Foot complication**					
	Yes	35 (0.5)	20 (57.1)	15 (42.9)	0.161
	No	7,611 (95.5)	3,449 (45.3)	4,162 (54.7)	
**Number of oral antidiabetic drugs**					
	None or lifestyle modification	144 (1.9)	136 (94.4)	8 (5.6)	<0.001
	Monotherapy	2,415 (31.6)	1,601 (66.3)	814 (33.7)	
	Dual or triple therapy	5,087 (66.5)	1,732 (34.0)	3,335 (66.0)	
**Antihypertensive medications**					
	Yes	5,927 (77.5)	2,642 (44.6)	3,285 (55.4)	0.010
	No	1,719 (22.5)	827 (48.1)	892 (51.9)	
**Lipid-lowering medications**					
	Yes	5,368 (70.2)	2,408 (44.9)	2,960 (55.1)	0.168
	No	2,278 (29.8)	1,061 (46.6)	1,217 (53.4)	
**Antiplatelet medications**					
	Yes	2,125 (27.8)	1,012 (47.6)	1,113 (52.4)	0.014
	No	5,521 (72.2)	2,457 (44.5)	3,064 (55.5)	
**Polypharmacy**					
	Yes	3,077 (40.2)	1,271 (41.3)	1,806 (58.7)	<0.001
	No	4,569 (59.8)	2,198 (48.1)	2,371 (51.9)	
**Baseline HbA1c**					
	7 –<8% (53 –<64 mmol/mol)	2,951 (38.6)	923 (31.1)	2,028 (68.7)	<0.001
	8 –<9% (64 –<75 mmol/mol)	1,729 (22.6)	760 (44.0)	969 (56.0)	
	≥9% (≥75 mmol/mol)	2,966 (38.8)	1,786 (60.2)	1,180 (39.8)	

IQR, interquartile range; SD, standard deviation

A significantly higher proportion of younger adults, Malays and obese patients received TI. The proportion of patients with TI was also higher among those on antiplatelet medications and with baseline HbA1c of ≥9% (≥75 mmol/mol). On the other hand, TI was less commonly observed in patients who had dual/triple OADs, patients who were on antihypertensive medications and those with polypharmacy.

### Time to treatment intensification

**Fig 2:**
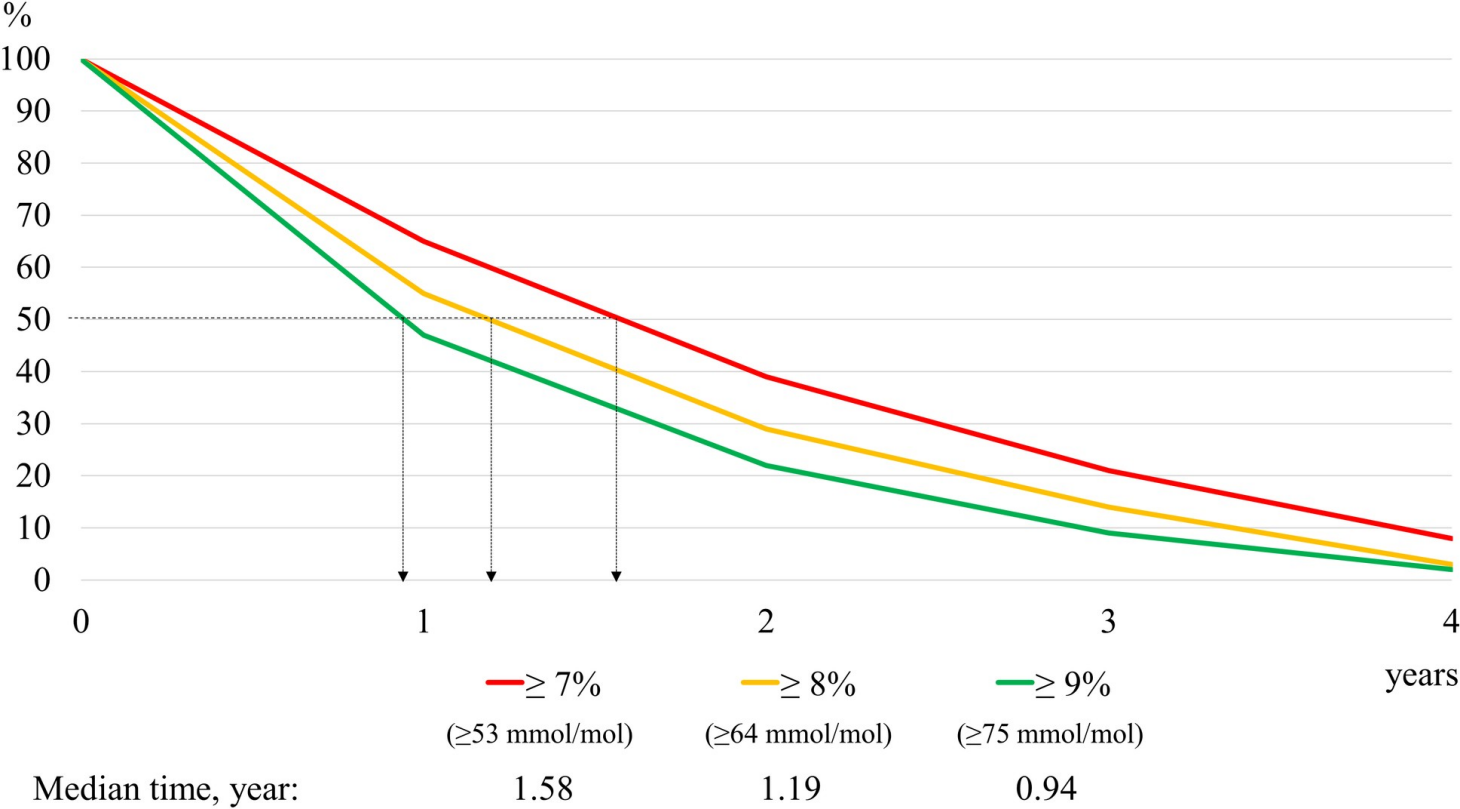
Survival curves for median time to treatment intensification using different HbA1c cutoffs.

The mean follow-up duration was 2.6 (SD 1.1) years with a cumulative follow-up of 20,151 person-years. Table 2 shows the median time to TI under different scenarios and HbA1c cutoffs. The median times to TI were 1.29 years (15.5 months), 1.58 years (19.0 months) and 2.32 years (27.8 months) under the best-, average- and worst-case scenarios respectively. The sensitivity analysis demonstrated that the median time to TI reduced when the HbA1c cutoffs increased. This pattern was consistently observed under all the scenarios. Fig 2 depicts the survival curves for different HbA1c cutoffs based on the average-case scenario.

**Table 2:** Median time to treatment intensification under different scenarios and HbA1c cutoffs.

HbA1c cutoffs		n	Best-case, year	Average-case, year	Worst-case, year
	≥7% (≥53 mmol/mol)	7,646	1.29	1.58	2.32
	≥8% (≥64 mmol/mol)	4,695	0.92	1.19	1.84
	≥9% (≥75 mmol/mol)	2,966	0.81	0.94	1.52

n, number of patients

### Proportion of patients with treatment intensification

The proportion of patients with TI in this study was 45.4% (95% CI: 44.2–46.5) as shown in Table 3. Under the average-case scenario, about two-third of patients had timely TI within one year. The probability of a T2D patient to have timely TI was 0.45, 0.35 and 0.24 under the best-, average- and worst-case scenarios respectively. As the HbA1c cutoffs increased, the proportion of patients with TI increased from 45.4% to 59.6% (Table 3). Similarly, the proportions of patients with timely TI also increased in all different scenarios.

**Table 3:** Proportion of patients with timely treatment intensification under different scenarios and HbA1c cutoffs.

HbA1c cutoffs		n	n (%) with TI	Proportion of patients with timely TI among those with TI		
				Best-case, %	Average-case, %	Worst-case, %
	≥7% (≥53 mmol/mol)	7,646	3,469 (45.4)	77.7	65.4	46.8
	≥8% (≥64 mmol/mol)	4,695	2,522 (53.7)	82.3	70.9	52.0
	≥9% (≥75 mmol/mol)	2,966	1,767 (59.6)	86.0	76.1	56.4

CI, confidence interval; n, number of patients; TI, treatment intensification

### Factors associated with treatment intensification

Table 4 shows the multivariate discrete-time proportional hazards model for TI. Younger age group, patients who were overweight, obese, those who use antiplatelet medication and patients with higher baseline HbA1c were significantly more likely to have TI. The baseline HbA1c of ≥9% (≥75 mmol/mol) was the strongest factor to be associated with TI in the model. Incremental number of OADs were associated with reducing likelihood for TI. Compared to patients without any pharmacological treatment, patients on dual or triple therapy were ten times less likely to receive TI.

**Table 4:** Multivariate proportional hazards model for treatment intensification, n = 7,581.

Characteristics		Wald Chi-square	Hazard ratio	95% CI	*P value*
**Age group–younger adults <60 years**		23.59	1.21	1.12–1.30	<0.001
**Body mass index class**					
	Underweight	0.29	0.89	0.60–1.34	0.589
	Normal weight		1.00		
	Overweight	19.61	1.23	1.12–1.34	<0.001
	Obese	30.35	1.31	1.19–1.44	<0.001
**Number of oral antidiabetic drugs**					
	None or lifestyle modification		1.00		
	Monotherapy	63.36	0.33	0.26–0.44	<0.001
	Dual or triple therapy	281.06	0.10	0.08–0.13	<0.001
**Antiplatelet medications–yes**		27.46	1.24	1.14–1.34	<0.001
**Baseline HbA1c**					
	7 –<8% (53 –<64 mmol/mol)		1.00		
	8 –<9% (64 –<75 mmol/mol)	128.27	1.79	1.62–1.98	<0.001
	≥9% (≥75 mmol/mol)	712.64	3.30	3.03–3.60	<0.001

CI, confidence interval

Bayesian Information Criterion (BIC): 578.1, Akaike’s Information Criterion (AIC): 508.8, Log likehood: -244.4

Number of cases excluded was 65 (0.9%) due to missing data for variable BMI.

### Sensitivity analysis: Individualized HbA1c target

Among 544 patients with individualized HbA1c ≥8% (≥64 mmol/mol), 53.1% (95% CI: 48.8–57.4) of them had TI. (S1 Table) The proportion of patients with TI was higher in younger adults, overweight/obese patients and those with baseline HbA1c of ≥9% (≥75 mmol/mol). In contrast, TI was less commonly observed in patients who had dual/triple OADs and those with polypharmacy. The median time to TI was 0.91 year (10.9 months), 1.12 years (13.4 months) and 1.72 years (20.6 months) under the best-, average- and worst-case scenarios respectively. Among patients with TI, the proportion of patients with timely TI was 84.1%, 73.7% and 52.6% under the best-, average- and worst-case scenarios respectively. In the adjusted model, BMI class, number of OADs and baseline HbA1c were independent factors associated with TI; which is similar to the primary analysis. (S2 Table) Overweight/obese patients and those baseline HbA1c of ≥9% (≥75 mmol/mol) were significantly more likely to have TI. On the other hand, patients on dual or triple therapy were almost two-third less likely to receive TI. The results by individualized HbA1c target were consistent with the primary findings of this study.

### Post-hoc analysis

Post-hoc life table analysis for median time to TI under the best-worst scenarios were 0.54 and 0.77 year (6.5 and 9.2 months) for lifestyle modification without OAD, 0.76 to 1.38 year (9.1 to 16.6 months) for monotherapy and 1.94 to 2.95 years (23.3 to 35.4 months) for dual or triple therapy. Compared to other ethnicities, a higher proportion of Chinese patients were older adults, male, had diabetes for more than ten years, had normal BMI, hypertension and stroke. (S3 Table) The use of antihypertensive and antiplatelet medications as well as polypharmacy were highest among Chinese patients. They also had the lowest of proportion of baseline HbA1c ≥9% (≥75 mmol/mol).

## Discussion

### Characteristics of patients

Characteristics of patients in this study were very similar to patients in the Malaysian National Diabetes Registry whereby there were more females, Malays, non-smokers, comorbid hypertension and dyslipidemia [6]. Their mean age and the median duration of diabetes also resembled those in the registry [6].

### Treatment intensification

Treatment recommendations for T2D patients are based on their existing treatment regimens and HbA1c values during routine clinic follow-up. The higher the HbA1c values, the greater number of antihyperglycemic agents (including insulin) are recommended to be added [3]. For example, a patient on lifestyle treatment with a HbA1c above 10% (86 mmol/mol) is recommended to be started on metformin, another OAD and insulin [3]. Hence, the definition of TI included an increase in the number of OAD or the initiation of insulin. In a systematic review, many studies defined TI as the addition of OAD or insulin/injectable to the index treatment [10].

### Time to treatment intensification

Our findings suggested that more than half of the patients had clinical inertia as the median time to TI was greater than one year even under the best-case scenario. Among patients with HbA1c above individualized target, the median time to TI was also beyond the one-year mark in the average-case scenario. Our results were consistent with a systematic review where the median time to TI was more than one year in nine out of ten studies in the US, UK and Spain [10]. Nevertheless, a study in Israel reported patients who failed metformin monotherapy had TI with a median time of 4.1 months only [25]. The time to TI decreased with increasing HbA1c thresholds was observed, similarly as reported in the systematic review [10]. This made clinical sense as the need for TI became more apparent as the HbA1c values worsen [3, 22].

### Proportion of patients with treatment intensification

Our study found 45.4% of patients had TI which was within the range of 37–79% reported in a systematic review [10]. Among patients with TI, about one-third had TI only after one year. It is worrying as the probability of a T2D patient to have timely TI is less than 50–50 chance, even under the best-case scenario. This occurred despite clinical recommendation urging for three monthly HbA1c investigations to enable timely treatment adjustment [3, 22]. Consistent with a systematic review, the proportion of our patients with TI increased with increasing HbA1c thresholds [10]. Among patients with HbA1c above individualized target, the proportion of patients with TI was also similar to other studies [10].

### Factors associated with treatment intensification

Ethnicity was a factor associated with TI in the univariate analysis. Notably, Chinese patients had the lowest proportion of TI among several ethnicities. However, ethnicity was not an independent factor for TI in the adjusted model. Ethnicity was most probably a confounding factor for age group, BMI class, antiplatelet medication and baseline HbA1c.

Younger adults were more likely to have TI and this finding was similarly across other studies [25–29]. Clinical practice guidelines have recommended for a more stringent HbA1c target in younger patients [3, 22]. An early good glycemic control would reduce vascular complications and mortality later on in the disease trajectory [1]. In the management of T2D among older adults, TI may not be clinically indicated in some of them who had individualized HbA1c target of <8–8.5% (<64–69 mmol/mol) due to their short life expectancy, advanced disease, comorbidities or complications [3, 22]. When we accounted for individualized HbA1c target, age group was not an independent factor associated with TI.

Patients who were overweight and obese had a greater likelihood for TI, similarly reported in other studies [25, 28, 30, 31]. Overweight or obesity is closely associated with glycemic control and is an independent risk factor for cardiovascular complications [2, 3]. We found no association between comorbidities and complications with TI, as reported in other studies [29–31]. We believe TI among patients with multiple commodities and complications is a delicate balance between risks and its benefits. While intensive glycemic control reduces the rates of non-fatal myocardial infarction, it increases the risk of severe hypoglycemia [32].

Similar with other studies, higher number of OADs decreased the likelihood for TI [26, 31]. The use of multiple medications can lead to low adherence, side effects and incur additional cost. A consensus report has recommended due consideration on using multiple combination of antidiabetic medications and to avoid overly burdensome regimens [2]. Our post-hoc analysis revealed that even under the best-case scenario, the median time to TI in patients with dual or triple OADs was nearly two years. On the other hand, patients who were not on any OADs received a timelier TI; the median time to TI was about nine months under the worst-case scenario.

Our findings demonstrated that higher HbA1c was positively associated with TI, similar with other studies [25, 27, 29–31]. In both the Malaysian and American clinical guidelines, the least stringent individualized HbA1c target was <8.0–8.5% (<64–69 mmol/mol) [3, 22]. The HbA1c value of ≥9% (≥75 mmol/mol) was too high, even for patients with short life expectancy and had multiple complex comorbidities and complications. Hence, TI was most likely to occur among this group of patients. Besides that, more TI among patients who used antiplatelet medication may be reflective of a better patient care among those at risk for cardiovascular complications because T2D confers about two-fold excess risk to develop cardiovascular disease [33].

Studies have shown that TI improved glycemic control [29] and patients receiving earlier TI will achieve their HbA1c goal sooner [27]. A large cohort study in the UK found that a one year delay of TI significantly increased risks of myocardial infarction, stroke and heart failure by 67%, 51% and 64% respectively [34]. Therefore, it is very important to address the issue of clinical inertia in our health settings.

There are several limitations in this study. Firstly, our definition for TI did not include dosage increment of a pre-existing OAD regimen. This was unavoidable as dosage information was not available in the NDR. Hence, we used one-year cutoff time to provide enough time for active dose titration to maximum dosage. Secondly, discrete-time data was used instead of continuous-time; this disallowed us to observe the exact time when TI occurred. We addressed this by using life table survival analysis to determine and compare median time to TI. Thirdly, the last laboratory and clinical results were used to represent the entire year performance which did not account for potential HbA1c fluctuations during the year. Fourthly, as with all registries, missing data were unavoidable. We addressed this by using best-worst case scenario analysis and were able to quantify measures of clinical inertia. Finally, some information were not studied such as patients’ health literacy level, visit consultation time and levels of physician care, all of which may affect TI [8].

To the best of our knowledge, this is among the first study in Malaysia to determine the time to TI in elucidating clinical inertia in T2D management. We contribute to the urgent need of such data from LMIC as highlighted by a systematic review [10]. By quantifying the measurement of clinical inertia and identifying the factors associated with TI, our results will enable the policy makers and clinicians to improve the quality of diabetes care. Our real-world clinical data reflect the daily practice and the findings may be applicable to other LMIC like Malaysia.

## Conclusions

Clinical inertia exists in the management of T2D patients in the Malaysia public health clinics. This is evidenced from the median time to TI at greater than one year, even under the best-case scenario. During the 2.6 years of follow-up, less than half of the patients had TI. The probability of a T2D patient with uncontrolled glycemia to have timely TI was less than 50%, even under the best-case scenario. Younger adults, overweight, obesity, use of antiplatelet medications and poorer HbA1c were positively associated with TI. Patients treated with more oral antidiabetics were less likely to have TI. When higher HbA1c cutoffs and individualized HbA1c target were used, albeit a better performance, our results still suggest the presence of clinical inertia.

As clinical inertia is a complex condition with clinician-, patient- and healthcare system-factors, we recommend further studies in LMIC to explore the causes so that targeted strategies can be developed and implemented. Prospective cohort study using continuous-time data can also be conducted to further delineate clinical inertia in T2D management.

## Supporting information

S1 Table Characteristics of patients with HbA1c above individualized target. (DOCX)

S2 Table Multivariate proportional hazards model for treatment intensification among patients with HbA1c above individualized target, n = 531. (DOCX)

S3 Table Characteristics of patients by ethnicity. (DOCX)
