# Supplementary material for: Clinical inertia in type 2 diabetes management in a middle-income country: A retrospective cohort study
Source: PLoS One. 2020 Oct 9;15(10):e0240531. doi: 10.1371/journal.pone.0240531 (PMC7546487; doi:10.1371/journal.pone.0240531)
Supplement: S1 Table — (DOCX) [file pone.0240531.s001.docx]

**S1 Table: Characteristics of patients with HbA1c above individualised target**

| **Characteristics** | | **Total**  **n (column %)** | **Treatment intensification** | | ***P value*** |
| --- | --- | --- | --- | --- | --- |
|  |  |  | **Yes, n (row %)** | **No, n (row %)** |  |
|  |  | **544 (100)** | **289 (53.1)** | **255 (46.9)** |  |
| **Age,** mean (SD) | | 67.0 (11.1) | 65.7 (10.8) | 68.5 (11.2) | 0.003 |
|  | Younger adults | 137 (25.2) | 82 (59.9) | 55 (40.1) | 0.068 |
|  | Older adults | 407 (74.8) | 207 (50.9) | 200 (49.1) |  |
| **Sex** | |  |  |  |  |
|  | Male | 247 (45.4) | 132 (53.4) | 115 (46.6) | 0.893 |
|  | Female | 297 (54.6) | 157 (52.9) | 140 (47.1) |  |
| **Ethnicity** | |  |  |  |  |
|  | Malay | 355 (65.3) | 199 (56.1) | 156 (43.9) | 0.103 |
|  | Chinese | 108 (19.9) | 48 (44.4) | 60 (55.6) |  |
|  | Indian | 80 (14.7) | 41 (51.2) | 39 (48.8) |  |
|  | Others | 1 (0.2) | 1 (100.0) | 0 (0.0) |  |
| **Duration of diabetes**, median (IQR) | | 9.0 (10.0) | 10.0 (11.0) | 9.0 (9.0) | 0.088 |
|  | <5 years | 114 (21.0) | 57 (50.0) | 57 (50.0) | 0.480 |
|  | 5 – 10 years | 200 (36.8) | 103 (51.5) | 97 (48.5) |  |
|  | >10 years | 230 (42.3) | 129 (56.1) | 101 (43.9) |  |
| **Smoker** | |  |  |  |  |
|  | Yes | 39 (7.2) | 23 (59.0) | 16 (41.0) | 0.447 |
|  | No | 505 (92.8) | 266 (52.7) | 239 (47.3) |  |
| **Body mass index**, kg/m^2^, mean (SD)  (n = 531 due to missing data) | | 26.7 (4.8) | 27.4 (5.0) | 25.9 (4.4) | <0.001 |
|  | Underweight, <18.5 | 7 (1.3) | 4 (57.1) | 3 (42.9) | 0.013 |
|  | Normal, 18.5 - <25.0 | 213 (40.1) | 98 (46.0) | 115 (54.0) |  |
|  | Overweight, 25 - <30.0 | 184 (34.7) | 101 (54.9) | 83 (45.1) |  |
|  | Obese, ≥30.0 | 127 (23.9) | 81 (63.8) | 46 (36.2) |  |
| **Hypertension** | |  |  |  |  |
|  | Yes | 521 (95.8) | 275 (52.8) | 246 (47.2) | 0.447 |
|  | No | 23 (4.2) | 14 (60.9) | 9 (39.1) |  |
| **Dyslipidemia** | |  |  |  |  |
|  | Yes | 490 (90.1) | 266 (54.3) | 224 (45.7) | 0.102 |
|  | No | 54 (9.9) | 23 (42.6) | 31 (57.4) |  |
| **Ischemic heart disease** | |  |  |  |  |
|  | Yes | 102 (18.8) | 63 (61.8) | 39 (38.2) | 0.052 |
|  | No | 442 (81.3) | 226 (51.1) | 216 (48.9) |  |
| **Stroke** | |  |  |  |  |
|  | Yes | 24 (4.4) | 13 (54.2) | 11 (45.8) | 0.917 |
|  | No | 520 (95.6) | 276 (53.1) | 244 (46.9) |  |
| **Nephropathy** | |  |  |  |  |
|  | Yes | 159 (29.2) | 87 (54.7) | 72 (45.3) | 0.633 |
|  | No | 385 (70.8) | 202 (52.5) | 183 (47.5) |  |
| **Retinopathy** | |  |  |  |  |
|  | Yes | 73 (13.4) | 44 (60.3) | 29 (39.7) | 0.188 |
|  | No | 471 (86.6) | 245 (52.0) | 226 (48.0) |  |
| **Foot complication** | |  |  |  |  |
|  | Yes | 19 (3.5) | 11 (57.9) | 8 (42.1) | 0.671 |
|  | No | 525 (96.5) | 278 (53.0) | 247 (47.0) |  |
| **Number of oral antidiabetic drugs** | |  |  |  |  |
|  | None or lifestyle modification | 8 (1.5) | 8 (100.0) | 0 (0.0) | <0.001 |
|  | Monotherapy | 149 (27.4) | 110 (73.8) | 39 (26.2) |  |
|  | Dual or triple therapy | 387 (71.1) | 171 (44.2) | 216 (55.8) |  |
| **Antihypertensive medications** | |  |  |  |  |
|  | Yes | 502 (92.3) | 264 (52.6) | 238 (47.4) | 0.387 |
|  | No | 42 (7.7) | 25 (59.5) | 17 (40.5) |  |
| **Lipid-lowering medications** | |  |  |  |  |
|  | Yes | 443 (81.4) | 236 (53.3) | 207 (46.7) | 0.885 |
|  | No | 101 (18.6) | 53 (52.5) | 48 (47.5) |  |
| **Antiplatelet medications** | |  |  |  |  |
|  | Yes | 241 (44.3) | 122 (50.6) | 119 (49.4) | 0.297 |
|  | No | 303 (55.7) | 167 (55.1) | 136 (44.9) |  |
| **Polypharmacy** | |  |  |  |  |
|  | Yes | 333 (61.2) | 162 (48.6) | 171 (51.4) | 0.009 |
|  | No | 211 (38.8) | 127 (60.2) | 84 (39.8) |  |
| **Baseline HbA1c** | |  |  |  |  |
|  | 8 – <9% (64 – <75 mmol/mol) | 218 (40.1) | 98 (45.0) | 120 (55.0) | 0.002 |
|  | ≥9% (≥75 mmol/mol) | 326 (59.9) | 191 (58.6) | 135 (41.4) |  |
