# Supplementary material for: Clinical inertia in type 2 diabetes management in a middle-income country: A retrospective cohort study
Source: PLoS One. 2020 Oct 9;15(10):e0240531. doi: 10.1371/journal.pone.0240531 (PMC7546487; doi:10.1371/journal.pone.0240531)
Supplement: S2 Table — (DOCX) [file pone.0240531.s002.docx]

**S2 Table:** **Multivariate proportional hazards model for treatment intensification among patients with HbA1c above individualized target, n = 531**

| **Characteristics** | | **Wald Chi-square** | **Hazard ratio** | **95% CI** | ***P value*** |
| --- | --- | --- | --- | --- | --- |
| **Body mass index class** | |  |  |  |  |
|  | Underweight | 0.90 | 1.65 | 0.59 – 4.64 | 0.343 |
|  | Normal weight |  | 1.00 |  |  |
|  | Overweight | 4.92 | 1.39 | 1.04 – 1.86 | 0.035 |
|  | Obese | 13.75 | 1.83 | 1.33 – 2.52 | <0.001 |
| **Number of oral antidiabetic drugs** | |  |  |  |  |
|  | None or monotherapy |  | 1.00 |  |  |
|  | Dual or triple therapy | 56.37 | 0.36 | 0.28 – 0.47 | <0.001 |
| **Baseline HbA1c** | |  |  |  |  |
|  | 8 – <9% (64 – <75 mmol/mol) |  | 1.00 |  |  |
|  | ≥9% (≥75 mmol/mol) | 13.32 | 1.63 | 1.25 – 2.12 | <0.001 |
| CI, confidence interval | | | | | |
| Bayesian Information Criterion (BIC): 95.8, Akaike’s Information Criterion (AIC): 70.1, Log likehood: -29.1 | | | | | |
| Number of cases excluded was 13 (2.4%) due to missing data for body mass index. | | | | | |
