# Supplementary material for: Clinical inertia in type 2 diabetes management in a middle-income country: A retrospective cohort study
Source: PLoS One. 2020 Oct 9;15(10):e0240531. doi: 10.1371/journal.pone.0240531 (PMC7546487; doi:10.1371/journal.pone.0240531)
Supplement: S3 Table — (DOCX) [file pone.0240531.s003.docx]

**S3 Table: Characteristics of patients by ethnicity**

|  | | **Chinese** | **Malays** | **Indians** | **Others** | *p-values* |
| --- | --- | --- | --- | --- | --- | --- |
|  |  | **n (column %)** | **n (column %)** | **n (column %)** | **n (column %)** |  |
|  |  | **1,149 (100)** | **5,010 (100)** | **1,444 (100)** | **43 (100)** |  |
| **Age,** mean (SD) | | 62.6 (10.5) | 57.7 (10.1) | 56.0 (10.1) | 52.4 (10.4) | <0.001 |
|  | Younger adults | 420 (36.6) | 2,877 (57.4) | 919 (63.6) | 33 (76.7) | <0.001 |
|  | Older adults | 729 (63.4) | 2,133 (42.6) | 525 (36.4) | 10 (23.3) |  |
| **Sex** | |  |  |  |  | <0.001 |
|  | Male | 542 (47.2) | 1,900 (37.9) | 563 (39.0) | 16 (37.2) |  |
|  | Female | 607 (52.8) | 3,110 (62.1) | 881 (61.0) | 27 (62.8) |  |
| **Duration of diabetes,** median (IQR) | | 5.0 (6.0) | 4.0 (5.0) | 5.0 (6.0) | 2.0 (3.0) | <0.001 |
|  | <5 years | 492 (42.8) | 2,608 (52.1) | 687 (47.6) | 34 (79.1) | <0.001 |
|  | 5 – 10 years | 412 (35.9) | 1,809 (36.1) | 516 (35.7) | 9 (20.9) |  |
|  | >10 years | 245 (21.3) | 593 (11.8) | 241 (16.7) | 0 (0.0) |  |
| **Smoker** | |  |  |  |  | <0.001 |
|  | Yes | 71 (6.2) | 312 (6.2) | 65 (4.5) | 9 (20.9) |  |
|  | No | 1,078 (93.8) | 4,698 (93.8) | 1,379 (95.5) | 34 (79.1) |  |
| **Body mass index**, kg/m^2^, mean (SD)  (n = 7,581 due to missing data) | | 26.5 (4.4) | 28.5 (5.3) | 27.8 (5.2) | 29.1 (5.7) | <0.001 |
|  | Underweight, <18.5 | 10 (0.9) | 49 (1.0) | 15 (1.0) | 0 (0.0) | <0.001 |
|  | Normal, 18.5 - <25.0 | 433 (38.1) | 1,236 (24.9) | 429 (29.9) | 11 (25.6) |  |
|  | Overweight, 25 - <30.0 | 471 (41.4) | 1,953 (39.3) | 586 (40.8) | 17 (39.5) |  |
|  | Obese, ≥30.0 | 223 (19.6) | 1,727 (34.8) | 406 (28.3) | 15 (34.9) |  |
| **Hypertension** | |  |  |  |  | <0.001 |
|  | Yes | 987 (85.9) | 4,075 (81.3) | 1,054 (73.0) | 32 (74.4) |  |
|  | No | 162 (14.1) | 935 (18.7) | 390 (27.0) | 11 (25.6) |  |
| **Dyslipidemia** | |  |  |  |  | <0.001 |
|  | Yes | 882 (76.8) | 3,901 (77.9) | 1,044 (72.3) | 31 (72.1) |  |
|  | No | 267 (23.2) | 1,109 (22.1) | 400 (27.7) | 12 (27.9) |  |
| **Ischemic heart disease** | |  |  |  |  | 0.694 |
|  | Yes | 33 (2.9) | 130 (2.6) | 38 (2.6) | 0 (0.0) |  |
|  | No | 1,116 (97.1) | 4,880 (97.4) | 1,406 (97.4) | 43 (100.0) |  |
| **Stroke** | |  |  |  |  | 0.021 |
|  | Yes | 16 (1.4) | 33 (0.7) | 6 (0.4) | 0 (0.0) |  |
|  | No | 1,133 (98.6) | 4,977 (99.3) | 1,438 (99.6) | 43 (100.0) |  |
| **Nephropathy** | |  |  |  |  | 0.226 |
|  | Yes | 57 (5.0) | 209 (4.2) | 49 (3.4) | 1 (2.3) |  |
|  | No | 1,092 (95.0) | 4,801 (95.8) | 1,395 (96.6) | 42 (97.7) |  |
| **Retinopathy** | |  |  |  |  | 0.706 |
|  | Yes | 26 (2.3) | 118 (2.4) | 30 (2.1) | 0 (0.0) |  |
|  | No | 1,123 (97.7) | 4,892 (97.6) | 1,414 (97.9) | 43 (100.0) |  |
| **Foot complication** | |  |  |  |  | 0.949 |
|  | Yes | 6 (0.5) | 23 (0.5) | 6 (0.4) | 0 (0.0) |  |
|  | No | 1,143 (99.5) | 4,987 (99.5) | 1,438 (99.6) | 43 (100.0) |  |
| **Number of oral antidiabetic drugs** | |  |  |  |  | <0.001 |
|  | None or lifestyle modification | 22 (1.9) | 95 (1.9) | 26 (1.8) | 1 (2.3) |  |
|  | Monotherapy | 347 (30.2) | 1,657 (33.1) | 388 (26.9) | 23 (53.5) |  |
|  | Dual or triple therapy | 780 (67.9) | 3,258 (65.0) | 1,030 (71.3) | 19 (44.2) |  |
| **Antihypertensive medications** | |  |  |  |  | <0.001 |
|  | Yes | 946 (82.3) | 3,940 (78.6) | 1,010 (69.9) | 31 (72.1) |  |
|  | No | 203 (17.7) | 1,070 (21.4) | 434 (30.1) | 12 (27.9) |  |
| **Lipid-lowering medications** | |  |  |  |  | 0.004 |
|  | Yes | 794 (69.1) | 3,582 (71.5) | 962 (66.6) | 30 (69.8) |  |
|  | No | 355 (30.9) | 1,428 (28.5) | 482 (33.4) | 13 (30.2) |  |
| **Antiplatelet medications** | |  |  |  |  | 0.006 |
|  | Yes | 366 (31.9) | 1,373 (27.4) | 376 (26.0) | 10 (23.3) |  |
|  | No | 783 (68.1) | 3,637 (72.6) | 1,068 (74.0) | 33 (76.7) |  |
| **Polypharmacy** | |  |  |  |  | <0.001 |
|  | Yes | 507 (44.1) | 2,065 (41.2) | 490 (33.9) | 15 (34.9) |  |
|  | No | 642 (55.9) | 2,945 (58.8) | 954 (66.1) | 28 (65.1) |  |
| **Baseline HbA1c** | |  |  |  |  | <0.001 |
|  | 7 – <8% (53 – <64 mmol/mol) | 549 (47.8) | 1,863 (37.2) | 522 (36.1) | 17 (39.5) |  |
|  | 8 – <9% (64 – <75 mmol/mol) | 296 (25.8) | 1,093 (21.8) | 329 (22.8) | 11 (25.6) |  |
|  | ≥9% (≥75 mmol/mol) | 304 (26.5) | 2,054 (41.0) | 593 (41.1) | 15 (34.9) |  |
